# Clinical efficacy of dienogest against endometriomas with a maximum diameter of ≥4 cm

**DOI:** 10.1080/07853890.2024.2402942

**Published:** 2024-09-26

**Authors:** Yan Huang, Deyu Zhang, Lei Zhang, Yingfang Zhou, Chao Peng

**Affiliations:** Department of Obstetrics and Gynecology, Peking University First Hospital, Beijing, China

**Keywords:** Dienogest, pharmacotherapy, endometriosis, endometriomas, adverse effects

## Abstract

**Objective:**

This prospective observational study aims to demonstrate the clinical efficacy of dienogest in treating endometriomas with a maximum diameter of ≥4 cm.

**Methods:**

Patients (*n* = 81) with endometriomas (diameter of ≥4 cm) were enrolled and administered orally with dienogest (2 mg daily) and followed up for a year [Ethical approval code: 2020 Research 343]. Further, the efficacy was determined by recording the largest diameter and volume of the endometriomas, uterine volume, endometrial thickness, and the largest diameter of uterine fibroids in the patients during 0, 6, and 12 months. The pain symptoms were assessed using the Numerical Rating Scale (NRS), and the side effects of medication were monitored. With the consent, some patients underwent routine blood tests, and serum hormone, as well as Anti-Müllerian Hormone (AMH) levels were measured.

**Results:**

The dienogest treatment resulted in a significant reduction of the maximum diameter of these cysts from 50.5 mm to 41 mm in 6 months and 34 mm in 12 months. In addition, the volume of the cysts significantly decreased from 37.8 ml from baseline to 18.5 ml in 6 months and 11.8 ml in 12 months. Among 26 subjects with ultrasonic signs of endometrial polyps, 92.3% of cases displayed no polyps after 12 months. No significant changes were observed in the size of uterine fibroids and AMH levels. The NRS score showed a decrease from an average of 6.6–1.2 in 12 months.

**Conclusion:**

Dienogest could effectively reduce the diameter and volume of endometriomas with a maximum diameter of ≥4 cm, improving anemia, as well as pain symptoms and preserving ovarian function.

## 1. Introduction

Endometriosis is often characterized by the presence of functional endometrial tissue clumps (glands and stroma) outside the lining and the myometrium of the uterine cavity. Based on 2017 statistics, the prevalent benign gynecological disorder affected approximately 176 million women globally [1]. Endometriosis is primarily manifested as intolerable pain, infertility, and pelvic masses. In response to ovulation, several physiological sacs naturally develop within the ovaries. The invasion of ovarian tissue by the endometrial ectopic lesions predisposes to the formation of endometriotic cysts, commonly referred to as endometriomas. A previous study indicated that 17%–44% of patients with endometriosis could develop endometriomas [2]. Nevertheless, there exists no international consensus on the recommended size of endometriomas for surgical intervention.

According to the Chinese guidelines for the diagnosis and treatment of endometriosis, the expert consensus states that endometriomas with an average diameter of ≥4 cm may be considered for surgical treatment [3]. However, it should be noted that surgical procedures for endometriomas could significantly alleviate pain, inevitably impairing the ovarian reserve function. Previous reports indicated that the Anti-Müllerian Hormone (AMH) levels could decrease by 44%, 35%, and 54% in the early (1–6 weeks), mid (2–6 months), and late (9–18 months) post-operative treatment periods, respectively [4]. Notably, the substantial reduction in the AMH levels and antral follicle counts could be more pronounced in patients with larger cysts, bilateral cyst removal, and repeat surgeries [5,6]. To this end, the pathological condition of endometriosis is a chronic inflammatory disease with a complex pathogenesis and elusive etiology. Although surgical treatment can reduce pain, conservative surgery fails to eradicate lesions comprehensively. Post-operatively, about 20% of patients within two years may experience symptom recurrence necessitating further surgery, with a five-year recurrence rate as high as 40%–50% [7]. Nonetheless, the continuous management of the therapeutic regimen post-surgery is often prescribed to sustain pain relief, improve quality of life, and reduce the risk of recurrence [8].

Among various therapeutics, dienogest, chemically referred to as estradiol valerate, is a fourth-generation synthetic progestogen categorized as a 19-nortestosterone derivative that falls within the estrane class. This molecule exhibits high affinity and selectivity towards progesterone receptors. In addition, the pharmacokinetics of deinogest include oral bioavailability of approximately 90% with a steady-state concentration achieved in two days of administration, plasma half-life of 10 h, and rapid metabolism with no cumulative effect in the body upon discontinuation. Dienogest acts through a central negative feedback mechanism to regulate the Hypothalamic-Pituitary-Ovarian (HPO) axis, directly inducing apoptosis in the ovarian follicular granulosa cells, thereby inhibiting follicular development. Moreover, basal luteinizing hormone and follicle-stimulating hormone levels remain largely unaffected. In some previous reports, the ultrasonographic examinations indicated the development of antral follicles while maintaining estrogen at a therapeutic threshold level of 39 pg/ml for endometrial ectopic lesions, with rapid resumption of ovulation post-discontinuation [9,10]. At lesion sites, dienogest could inhibit the binding activity of NF-κB to DNA, reducing the expression levels of aromatase, COX-2, and PGE2. In addition, it could diminish ectopic lesion angiogenesis and promote apoptosis, effectively inhibiting the growth of endometriotic lesions [11–13]. Notably, dienogest lacks androgenic properties and possesses approximately 40% of the antiandrogenic activity of Cyproterone Acetate. Considering no glucocorticoid or anti-mineralocorticoid effects, the long-term use of fascinating molecule exerts minimal impact on the metabolic system (including carbohydrate metabolism, lipid metabolism, and coagulation function), cardiovascular system, and bone density, indicating a high safety profile for prolonged usage [10,14].

Dienogest has been approved by the European Regulatory Authorities in 2009 for the treatment of endometriosis and introduced to the Chinese market in 2019. Accordingly, several studies demonstrated the efficacy in alleviating pain associated with endometriosis, reducing lesion size, and its favorable safety profile [15,16]. Motivated by these aspects, this article aims to focus on exploring the clinical efficacy of dienogest in treating patients with cysts (diameter of > 4 cm). In this regard, we initially evaluate improved changes in cyst size and monitor parameters like uterine volume, endometrium, diameter of uterine fibroids, and AMH levels. In summary, we believe that this study provides real-world clinical data from China on the treatment of larger endometriomas with dienogest.

## 2. Experimental section

### 2.1. Design

Patients who visited the gynecological outpatient clinic at Peking University First Hospital from September 2021 to May 2022 and were willing to use dienogest for treating endometriomas were enrolled in this prospective study. This study was approved by the Ethics Committee of Peking University First Hospital (Ethical approval code: 2020 Research 343). The inclusion criteria were set as follows: Patients who underwent clinical diagnosis of endometriosis with ultrasonographic evidence of unilateral or bilateral endometriomas (a minimum diameter of 4 cm for any cyst); Patients aged between 18 and 50 years; Patients with regular menstrual cycles. Patients who refused surgery and showed no malignant signs, such as the presence of solid components, rich blood flow, or rapid growth. To this end, the exclusion criteria were set as follows: Patients who were planning to conceive; Patients who had used hormonal drugs or GnRH-a affecting hormone secretion within three months prior to the treatment; Patients with severe internal comorbidities, such as pelvic inflammatory diseases, malignant tumors, contraindications to dienogest; Patients who declined to participate in this observational study. All patients signed a written informed consent form before enrollment in this study.

### 2.2. Subjects

In this observational study, 81 patients were enrolled and were administered an oral daily dose of 2 mg of dienogest (Bayer, Visanne) and followed up for one year. The transvaginal ultrasonography was performed during the pre-determined intervals before treatment and after 6 and 12 months of treatment to assess the size of the endometriomas. Further, the maximum diameter of the cysts was measured [17,18], along with three dimensions (D1, D2, and D3) longitudinally, transversely, and anteroposteriorly of both the cysts and the uterus. Then, the volumes of the cysts and uterus were calculated using the ellipsoid formula: D1 × D2 × D3 × π/6. The endometrial thickness and the maximum diameter of uterine fibroids in patients with concomitant fibroids were also measured. The pain severity was assessed using the Numeric Rating Scale (NRS), and the side effects of the medication (vaginal bleeding, weight gain, headaches, decreased libido, acne, hair loss, and breast pain), as well as drug tolerance, were evaluated through questionnaires. In some cases, patients were suggested to undertake routine blood tests, hormone levels, and AMH assessments as per their consent.

### 2.3. Statistical analysis

Data not conforming to a normal distribution were presented using quartiles and box plots. The changes in diameter and volume of the cysts were analyzed using the non-parametric Wilcoxon signed-rank test for paired samples. The maximum diameter of the cysts was categorized into four groups (4–5 cm, 5–6 cm, 6–7 cm, and 7–8 cm) to compare differences in the changes in volume and diameter among these groups using the Kruskal–Wallis test for multiple independent samples. The relationship between the degree of reduction in cyst diameter and volume and the characteristics of the cysts (multilocular or unilocular, left or right side, unilateral or bilateral) was evaluated using the non-parametric Mann–Whitney *U* test for two independent samples. In addition, the correlation between the reduction in uterine volume and the presence of adenomyosis was evaluated using the Mann–Whitney *U* test. The changes in the endometrial thickness, uterine volume, maximum diameter of uterine fibroids, AMH levels, estrogen levels, and hemoglobin concentration were statistically analyzed using the non-parametric Wilcoxon signed-rank test for paired samples.

## 3. Results

### 3.1. The observational study presents the baseline characteristics of subjects

The observational study initially included 81 patients with cysts with a maximum diameter ranging from 4 to 8.2 cm. However, Three patients discontinued their participation due to depression after 1 month, reproductive need after 3 months, and personal reasons after 1 month, respectively. In total, there was a successful follow-up of 78 participants. The baseline characteristics and clinical features of these enrolled patients are presented in Table 1. During this observational study, 11 patients were transitioned to surgical treatment (Table 2), in which 9 of them underwent surgery between 3 and 9 months of medication. Accordingly, a total of 69 patients continued medication for one year (including 2 who underwent surgery after a year of medication) and were included in the statistical analysis. Out of these 69 patients, cysts with a maximum diameter of less than 4 cm were excluded, resulting in a total of 74 cysts (43 from the left ovary and 31 from the right ovary) being considered for analysis.

**Table 1.** A summary presents the demographic and clinical characteristics of the 78 patients successfully followed up.

Parameter	Value
Age (years)	32.9 ± 6.5
BMI	21.6 ± 3.8
Bilateral cysts (%)	35.9% (28/78)
Left-Sided cysts^a^ (%)	76.9% (60/78)
Right-Sided cysts^b^ (%)	59.0% (46/78)
Unilateral Left-Sided cysts^c^ (%)	41% (32/78)
Unilateral Right-Sided cysts^d^ (%)	23.1% (18/78)
Multilocular cysts (%)	41% (32/78)
Coexisting adenomyosis (%)	21.8% (17/78)
Coexisting uterine fibroids (%)	25.6% (20/78)
Coexisting endometrial polyps (%)	33.3% (26/78)

^a^Number of patients with left-sided endometriomas among the 78 patients, including those with only left-sided and bilateral cysts.

^b^Number of patients with right-sided endometriomas among the 78 patients, including those with only right-sided and bilateral cysts.

^c^Number of patients with only left-sided endometriomas among the 78 patients.

^d^Number of patients with only right-sided endometriomas among the 78 patients.

**Table 2.** A summary presents the duration of medication and the reasons why patients transitioned to surgery.

Patient	Duration of medication (months)	Reason
Patient 1	3	The unilateral cyst enlarged from 8.2 cm to 8.6 cm.
Patient 2	3	The unilateral cyst increased from 5.7 cm to 8 cm, and encapsulated effusion was discovered during surgery.
Patient 3	3	The unilateral cyst grew from 5.9 cm to 6.9 cm.
Patient 4	3	The unilateral cyst reduced from 5.7 cm to 4.7 cm in length, but pain relief was incomplete. Post-surgical pathology indicated a mucinous cystadenoma with an endometriosis background.
Patient 5	6	The bilateral cysts increased from 4.1 and 4.4 cm to 4.3 and 4.8 cm, respectively.
Patient 6	6	The unilateral cyst remained stable, changing from 6.4 cm to 6.5 cm
Patient 7	6	The unilateral cyst grew from 4 cm to 5 cm.
Patient 8	6	The unilateral cyst decreased from 4.7 cm to 4.3 cm, with unsatisfactory pain control.
Patient 9	9	The bilateral cysts increased from 3.8 and 5.1 cm to 4 cm and 5.2 cm, respectively, with the patient expressing a desire for fertility.
Patient 10	12	The unilateral cyst reduced from 4.7 cm to 4.3 cm, with the patient requesting surgery.
Patient 11	12	The unilateral cyst decreased from 5.6 cm to 3.4 cm, and encapsulated effusion was discovered, leading to surgery.

### 3.2. Dienogest alleviates pain symptoms

Among the enrolled 69 patients that were included in the statistical analysis, it was observed that dysmenorrhea was the most common pain symptom, with a few experiencing dyspareunia, chronic pelvic pain, and pain during defecation. Accordingly, the dienogest treatment significantly alleviated these pain symptoms. Moreover, several patients (*n* = 54) experienced varying degrees of dysmenorrhea, with an average Numeric Rating Scale (NRS) score of 6.6. After 6 and 12 months of dienogest treatment, the experimental results of an average NRS score dropped to 0.1, indicating a substantial relief in dysmenorrhea. In addition, several patients (*n* = 5) reported dyspareunia symptoms, with an average initial NRS score of 3 before treatment, which reduced to 0.4 in 6 and 12 months of dienogest treatment. Patients (*n* = 4) who experienced chronic pelvic pain with an initial NRS score of an average of 2.75 showed a decrease in its level to 0.5 after 6 and 12 months of dienogest treatment. In addition, four patients reported mild pain during defecation with an initial average NRS score of 1.75, which was lowered to 0.75 after dienogest treatment.

### 3.3. Dienogest reduces the maximum diameter and volume of endometriomas

**Figure 1.**
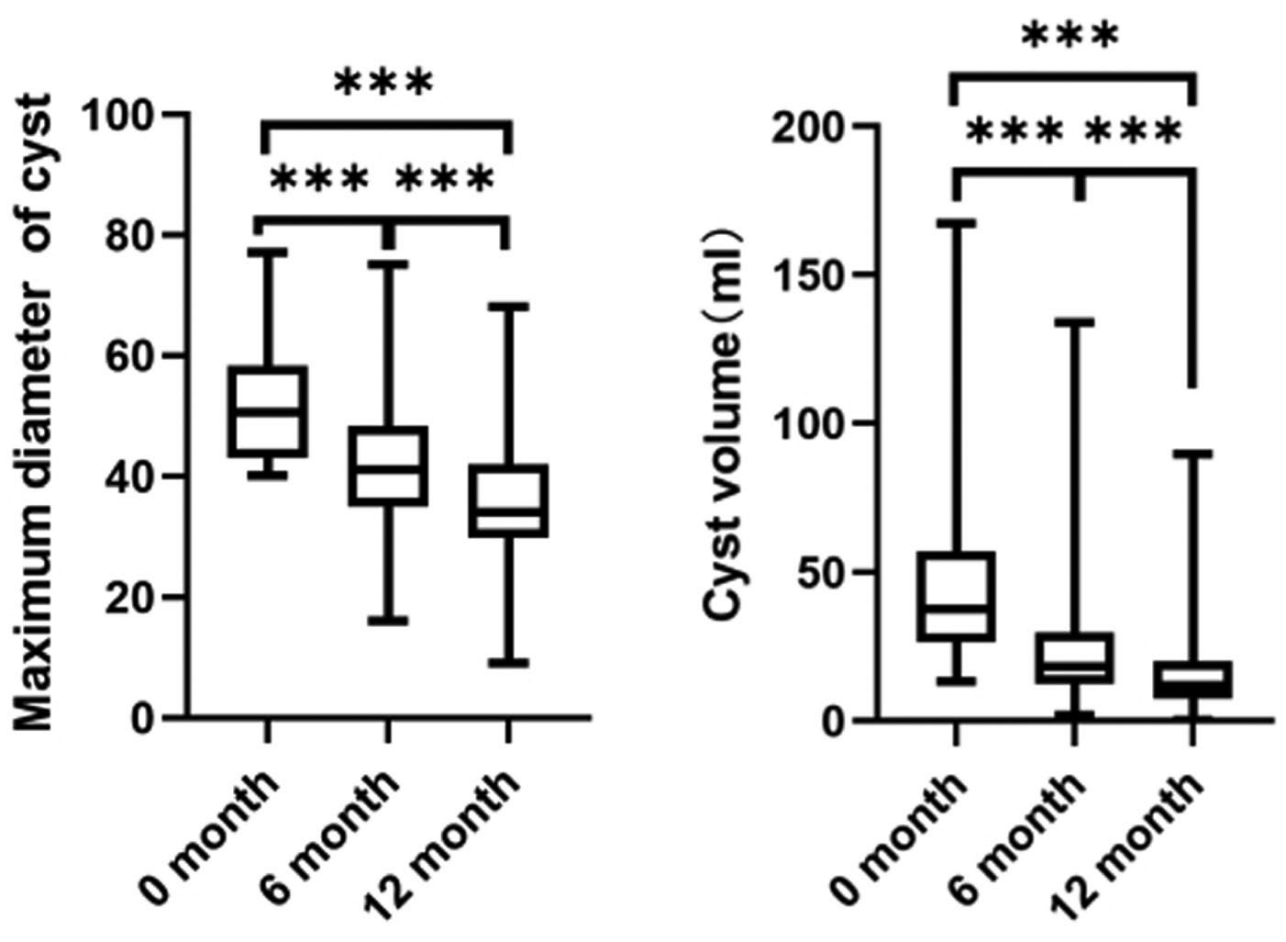
The image shows the changes in maximum diameter ((a), *n* = 74) and volume ((b), *n* = 74) of endometriomas treated with dienogest. The study evaluates 74 cases of endometriomas with an initial maximum diameter of ≥4 cm. Significant changes are observed in both the maximum diameter and volume of the cysts following dienogest treatment (*** indicates *p* < .001).

**Figure 2.**
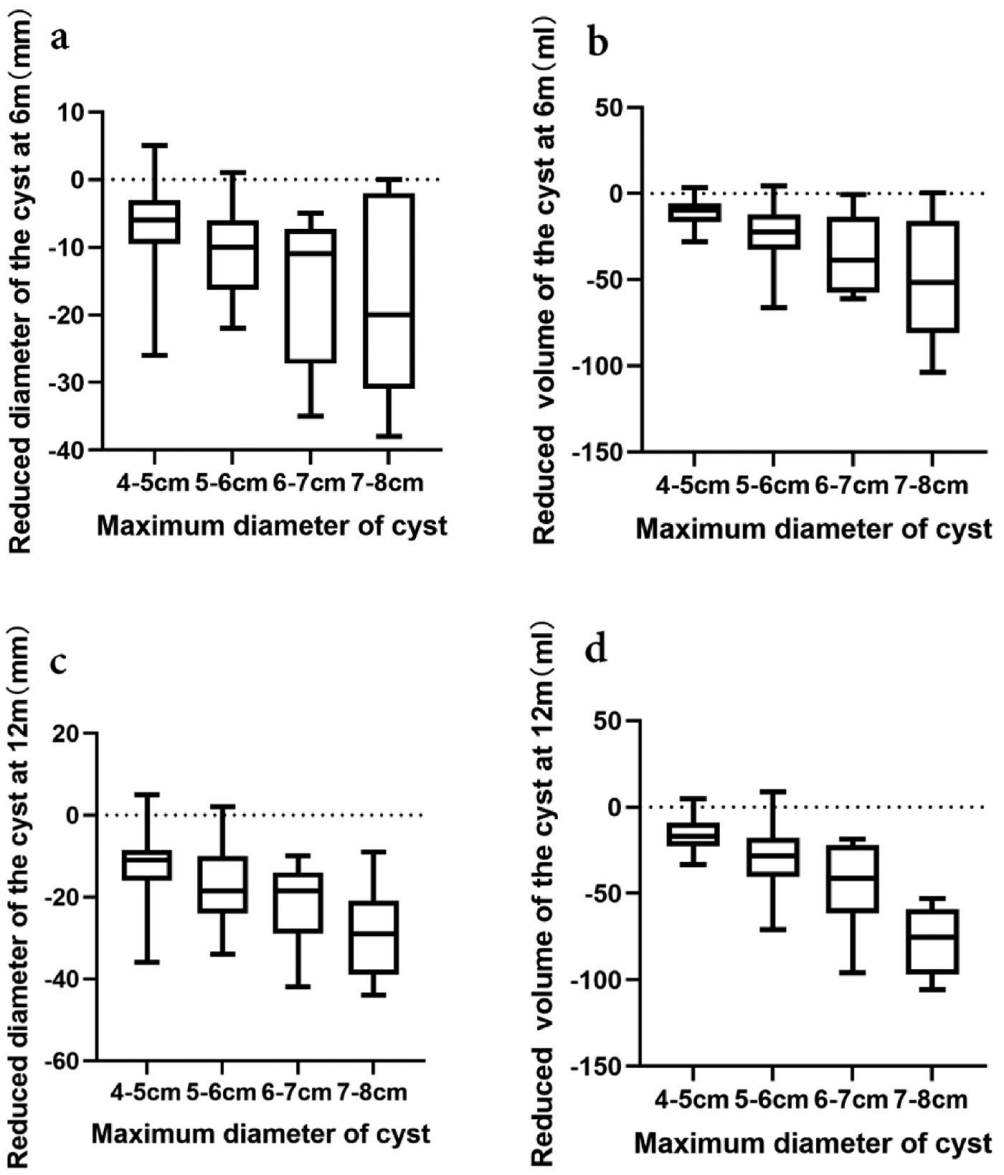
The data present the variation in the reduction of maximum diameter and volume of cysts of different diameters during the dienogest treatment. Cysts are categorized into four groups based on their maximum diameter: 33 cases in the 4–5 cm group, 26 cases in the 5–6 cm group, 8 cases in the 6–7 cm group, and 7 cases in the 7–8 cm group. After 6 months of treatment, a notable reduction is observed in the maximum diameter of the cysts ((a) intergroup difference *p* = .007) and cyst volume ((b) intergroup difference *p* = .000). After 12 months, there are significant reductions in both the maximum diameter ((c) intergroup difference *p* = .003) and volume ((d) intergroup difference *p* = .000) of the cysts.

Among the 69 enrolled patients after one year of treatment, 74 endometriomas with a maximum diameter of ≥4 cm were evaluated, including 43 from the left ovary and 31 from the right. The maximum diameter of these cysts before treatment was 50.5 [43, 58.3] mm, which reduced to 41 [35, 48.3] mm in 6 months and further to 34 [29.8, 42] mm in 12 months. In addition, the volume of the cysts decreased from 37.8 [26.6, 57.3] ml at baseline to 18.5 [12.5, 29.7] ml in 6 months and to 11.8 [7.5, 20.1] ml in 12 months. The experimental results indicated a significant reduction in the maximum diameter and volume of the cysts with dienogest treatment, as illustrated in Figure 1. Furthermore, the cysts were categorized into four different groups based on their maximum diameter, including 33 cases in the 4–5 cm group, 26 in the 5–6 cm group, 8 in the 6–7 cm group, and 7 in the 7–8 cm group. It was observed that larger cysts demonstrated a greater reduction in diameter and volume during dienogest treatment, with significant differences among the groups, as shown in Figure 2. Together, these findings indicated no correlation between the degree of reduction in cyst diameter and volume and the characteristics of the cysts (multilocular or unilocular, left or right side, unilateral or bilateral).

### 3.4. Dienogest impacts uterine volume, endometrium, and uterine fibroids

**Figure 3.**
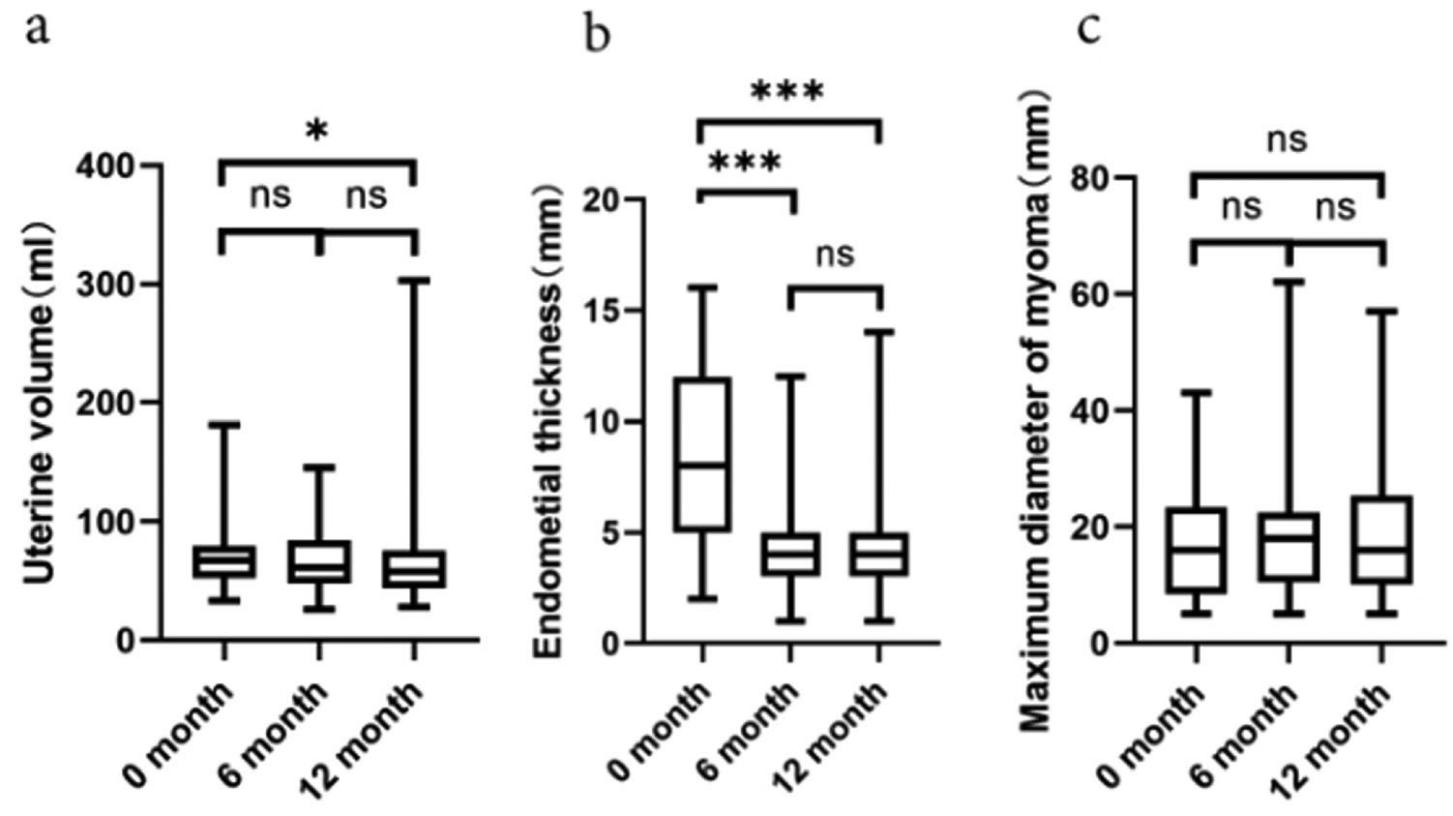
The data present changes in uterine volume, endometrial thickness, and maximum diameter of uterine fibroids during the dienogest treatment. After one year of dienogest treatment, changes are recorded in the uterine volume ((a), *n* = 69), endometrial thickness ((b), *n* = 69), and the maximum diameter of uterine fibroids ((c), *n* = 17). The results indicate no significant change (ns refers to *p* > .05), a minor change (* indicates *p* < .05), and a highly significant change (*** represents *p* < .001), respectively.

After a one-year treatment period with dienogest, a trend of reduction in terms of uterine volume was observed among the 69 patients. As shown in Figure 3a, the initial uterine volume before dienogest treatment was 67.1 [51.7 and 79.8] ml, which decreased to 60.9 [47.8 and 84.8] ml in 6 months and further to 58.3 [43.4 and 75.2] ml in 12 months of dienogest treatment. Despite an overall decrease in the uterine volume, 36.2% (25/69 cases) of the patients exhibited an increase in the uterine volume compared to baseline, with an average increase of 22.9% [9.3% and 44.2%]. The extent of reduction in the uterine volume was not correlated with the presence of adenomyosis. Moreover, a significant decrease in endometrial thickness was observed in the patients throughout the dienogest treatment. In addition, the average thickness of endometrium reduced from 7.5 [4.8 and 11.7] mm at baseline to 3.9 [3.1 and 5.0] mm in 6 months and to 3.6 [3.1 and 4.8] mm in 12 months of dienogest treatment (Figure 3b). Among the enrolled patients (*n* = 26) with endometrial polyp-like changes by ultrasonographic evidence, 80.8% (21/26) of them showed resolution of these changes in 6 months of dienogest treatment, and 92.3% (24/26) in 12 months of dienogest treatment. As illustrated in Figure 3c, no significant changes were observed in the maximum diameter of the fibroids throughout the treatment in the 17 patients with co-existing uterine fibroids.

### 3.5. Dienogest normalizes AMH, CA125, estrogen, and hemoglobin levels

**Figure 4.**
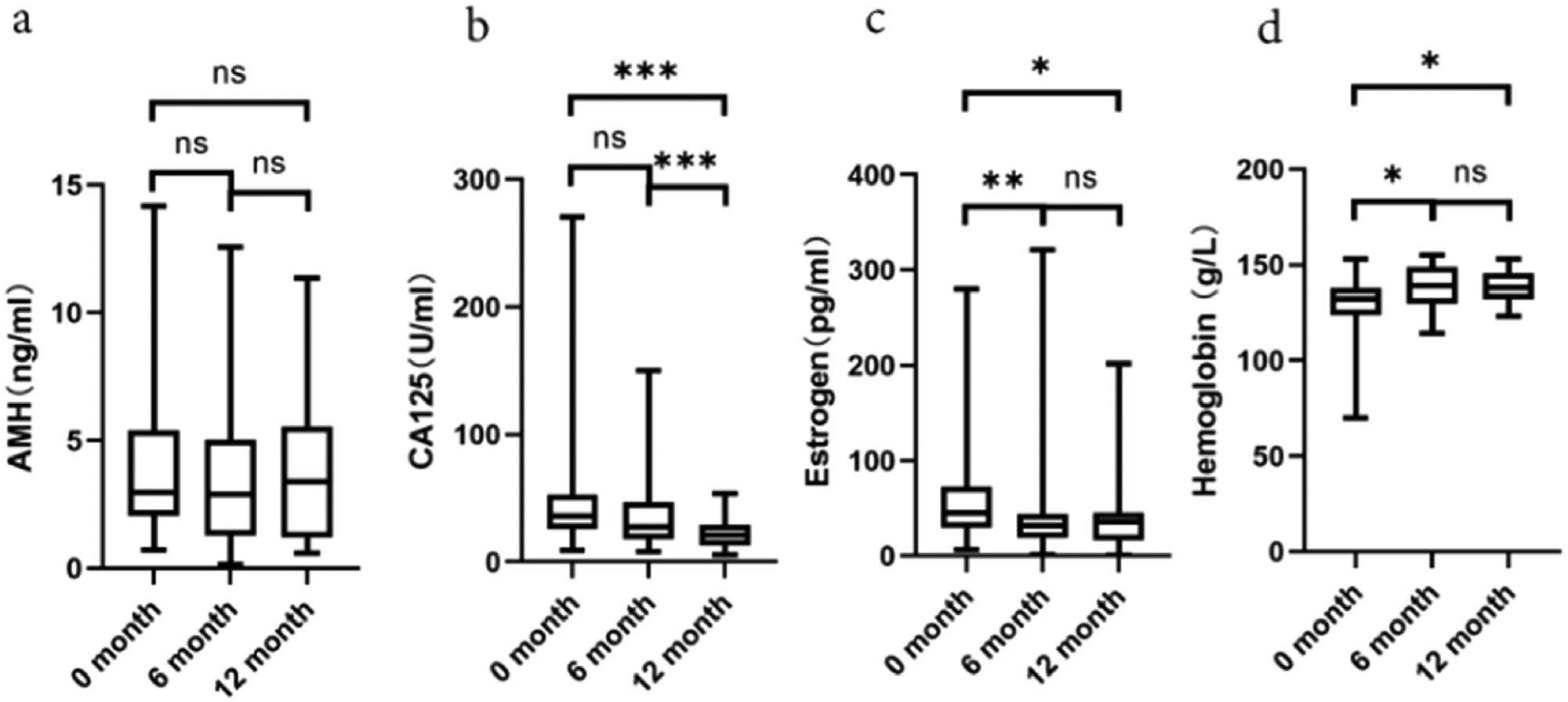
The data present the changes in AMH, CA125, estrogen, and hemoglobin concentration levels during the dienogest treatment. Throughout the 12-month dienogest treatment, there is no change in AMH levels (a, *n* = 36). A gradual decline is observed in CA125 levels (b, *n* = 46). Estrogen levels significantly decreased in the first 6 months and then stabilized (c, *n* = 39). An increase in hemoglobin concentration is noted, indicating a correction of anemia (d, *n* = 34). The statistical significance is marked as ns for no significance, * indicates *p* < .05, ** presents *p* < .01, and *** demonstrates *p* < .001.

During the dienogest treatment process in a year, no significant changes in the AMH levels were observed (Figure 4a). As depicted in Figure 4b, a progressive decline in the concentration of cancer antigen 125 (CA125) was observed, becoming increasingly pronounced with the elongation of the dienogest treatment duration. In addition, the experimental results indicated significantly decreased estrogen levels within the first 6 months of dienogest treatment, subsequently stabilized in 12 months (Figure 4c). Prior to dienogest treatment initiation, patients (*n* = 3) presented with mild to moderate anemia. Nevertheless, after 6 and 12 months of dienogest treatment, a marked elevation in the hemoglobin concentration was observed, normalizing the hemoglobin levels in the anemic patients (Figure 4d).

### 3.6. Dienogest exhibits tolerable adverse effects

Finally, the role of dienogest treatment in terms of safety was observed by determining the tolerable adverse effects. Among the 69 patients studied, 37.7% (26 out of 69) of cases experienced an increase in body weight, indicating the most common side effect of the dienogest treatment. It was observed from the results that the average weight gain was 1.96 ± 2.9 kg after 6 months and 2 ± 3.2 kg after 12 months of dienogest treatment. At the 6- and 12-month intervals, the condition of amenorrhea was reported in 84.1% (58 out of 69) and 88.4% (61 out of 69) of the cases, respectively. However, the remaining patients experienced varying degrees of spotting, which was found to be tolerable. Several miscellaneous adverse effects included breast tenderness in 17.4% (12 out of 69), mood depression in 20.3% (14 out of 69), hair loss in 14.5% (10 out of 69), headaches in 5.8% (4 out of 69), acne in 2.9% (2 out of 69), and reduced libido in 1.4% (1 out of 69) of the enrolled patients. Together, these findings suggested a high satisfaction rate with the treatment outcome, with 91.3% of the enrolled patients expressing satisfaction and willingness to continue the long-term dienogest treatment, indicating the tolerable side effects.

## 4. Discussion

Typically, endometriosis has been acknowledged in gynecology as a ‘benign cancer’. Although they are benign, the lesions exhibit extensive infiltrative growth in the pelvic cavity, commonly proliferating in areas such as the uterosacral ligaments, pelvic peritoneum, and ovaries [17]. In this observational study, we revealed a predilection for endometriomas to develop on the left side, which is a phenomenon whose underlying mechanism remained elusive. It could be hypothesized that this asymmetry might be linked to the leftward deviation of the sigmoid colon in the pelvis, influencing the anatomical alignment of the rectum and consequently predisposing the left pelvic cavity to accumulate retrograde menstrual flow.

Recently, dienogest has been recognized as an effective treatment for pain associated with endometriosis. Several potential side effects include irregular vaginal bleeding, weight gain, headaches, acne, hair loss, mood swings, and breast tenderness [18]. Similarly, this study identified weight gain as the most prevalent adverse reaction of dienogest treatment for a year. It has been increasingly demonstrated that progestogens could increase appetite, likely contributing to weight gain during the dienogest treatment. Prior to the treatment commencement, patients were informed about the possible side effects of the dienogest treatment. Throughout the treatment, the survey questionnaire responses from the majority of the enrolled patients suggested tolerability to the side effects, indicating the long-term use of the drug.

The pathogenesis of endometriosis is often characterized by its complexity and the difficulty in eradicating its causes. Despite the availability of conservative surgical interventions, they fail to remove all the lesions, requiring sustained chemotherapeutic management post-surgery to alleviate pain, improve quality of life, and lower the risk of recurrence [8]. The current study aimed at exploring the therapeutic efficacy of dienogest on patients who have no desire for parenthood. In the case of patients with fertility needs, they were given the option of dydrogesterone treatment [18,19]. Currently, there are no globally accepted guidelines regarding the size of endometriomas that warrant surgical therapeutic intervention. The Chinese guidelines, derived from expert consensus, recommend considering surgery for cysts measuring an average diameter of ≥4 cm [3]. However, the notified threshold value of ≥4 cm raises questions about the appropriate size of endometriomas for initiating pharmacological treatment *vs.* surgical intervention followed by chemotherapeutic treatment. These considerations led our team of researchers to undertake this research. Moreover, literature indicated that the larger cysts could be associated with a risk of spontaneous rupture, at a rate ranging from 2.6% to 9.0% [Dai, 2015 #1792; Gu, 2023 #1793]. The rupture exacerbates intra-pelvic inflammation and adhesion, possibly justifying surgical treatment for large-sized cysts. In a case, Dai and colleagues demonstrated that the critical diameter for distinguishing between ruptured and non-ruptured groups (*n* = 43 *vs.* 70) was 9.5 cm [20]. Notably, our current study indicated no instances of cyst rupture during a one-year follow-up period. Importantly, it should be noted that the risk of cyst rupture under long-term medication management could not be extensively quantified. Nevertheless, this risk could be reduced in larger cysts under pharmacological treatment, which might remain to be further investigated. In addition, the concern that medication might not effectively reduce the size of larger cysts was another factor influencing the decision to opt for surgical intervention. Recently, some studies indicated that the long-term dienogest treatment showed a decrease in the diameter and volume of endometriomas [15,21–23]. In an instance, Lee and coworkers revealed no significant difference in the treatment outcomes for cysts smaller or larger than 4 cm [22]. In our current study, dienogest treatment significantly reduced the diameter and volume of cysts with a maximum diameter of ≥4 cm, with a further reduction observed over time. As the cyst size increased, the degree of reduction in both diameter and volume also increased, suggesting that pharmacological (dienogest) treatment for cysts with a diameter of ≥ 4 cm could achieve satisfactory outcomes. Together, these findings provided literature support in terms of the chemotherapeutic management of larger endometriomas, potentially sparing several patients with cysts measuring ≥4 cm in diameter from the risks associated with surgical intervention, such as diminished ovarian function and other related complications. Moreover, the chemotherapeutic intervention could afford younger patients more opportunities for conservative treatment, allowing for an optimally timed single surgical procedure at required time intervals (e.g. for fertility purposes). Therefore, this approach could avoid repetitive surgeries or obviating the need for surgery altogether, thereby preserving ovarian function, enhancing fertility, and improving the quality of life of the patients.

The coexistence of endometriosis with estrogen-dependent diseases, such as adenomyosis, uterine fibroids, and endometrial polyps, is commonly observed. Notably, a comprehensive evaluation and integrated treatment approaches should be employed in such cases. However, there is a lack of reports on the follow-up of uterine volume, fibroids, and endometrial changes during the treatment. Previous reports indicated changes in the uterine volume in adenomyosis patients treated with dienogest. In an instance, Osuga and colleagues demonstrated that the average pre-treatment uterine volume in adenomyosis patients was 105.4 ml, which was significantly reduced by 20.6 ml in 24 weeks and 26.0 ml in 52 weeks post-treatment with dienogest [24]. In another case, Hirata and colleagues presented a decreasing trend in uterine volume (from 285.4 cm^3^ to 259.8 cm^3^) in adenomyosis patients treated with dienogest for 24 weeks, although the difference was statistically insignificant [25]. Fawzy and coworkers showed no significant reduction in the uterine volume in adenomyosis patients treated with dienogest for 16 weeks, with pre- and post-treatment volumes being 269 ± 157 ml and 236 ± 128 ml, respectively [26]. Accordingly, our study indicated a reduction in the uterine volume after 12 months of dienogest treatment. However, 36.2% of patients exhibited an increase in uterine volume compared to the baseline characteristic before treatment.

Moreover, no correlation was found between the degree of reduction in the uterine volume and the presence of adenomyosis. These findings suggested that the effect of dienogest on reducing uterine volume was limited. Similarly, the dienogest treatment could not appear to reduce the uterine fibroids. Moreover, the ultrasound investigations revealed a high incidence rate (37.7%) of endometrial polyps in patients with endometriosis. The endometrial polyps, a type of endometrial hyperplastic disease, are responsive to the high progestin effect of dienogest. Accordingly, our results confirmed that most patients experienced thinning of the endometrium and disappearance of polyps within 12 months of dienogest treatment, indicating that patients with ultrasound-identified endometrial polyps, could directly commence the pharmacological therapy, especially younger patients without high-risk factors or signs of endometrial pathology. However, unless there are indications of potential malignant transformation of the endometrium, pre-treatment polypectomy is not necessary. These findings could urge clinicians to reconsider the necessity of surgery in the treatment process, potentially saving more patients from the risks associated with hysteroscopic surgery.

Our study demonstrated that dienogest not only reduces the diameter and volume of endometriomas but also significantly alleviates pain symptoms. The treatment effectively reduces symptoms of heavy menstrual bleeding, corrects anemia, and increases hemoglobin concentration, which enhances patients’ overall quality of life. The observed stability of AMH levels during treatment indicates a protective effect on ovarian reserve function, which is crucial for preserving fertility in women of childbearing age. Although the surgical treatment could be more direct and effective, its impact on ovarian reserve function remained a major concern for women of childbearing age. In the post-laparoscopic cystectomy, a notable decrease in the AMH levels and antral follicle count was observed [4–6]^.^ Consisent with previous research findings [23], these findings suggested that dienogest showed a protective effect on ovarian reserve function. Throughout the treatment, a progressive decline in CA125 concentration was observed, becoming increasingly pronounced with prolonged treatment duration.This reduction in CA125, a biomarker often elevated in endometriosis, underscores the therapeutic impact of dienogest on disease activity [27]. Additionally, estrogen levels decreased significantly within the first six months of treatment and stabilized by twelve months. The decline in estrogen levels may contribute to symptom relief by mitigating the estrogen-dependent proliferation of endometrial tissue [28]. The marked elevation in hemoglobin concentrations after 6 and 12 months of treatment indicates a successful correction of anemia in patients who initially presented with mild to moderate anemia [29]. This improvement reflects dienogest’s ability to reduce menstrual blood loss and enhance the overall hematologic profile of patients.Mechnically, dienogest enhances autophagy induction in endometriotic cells by impairing activation of AKT, ERK1/2, and mTOR [30]^.^ Autophagy, a cellular degradation process that removes damaged organelles and proteins, is crucial for maintaining cellular homeostasis. By promoting autophagy, dienogest may contribute to the reduction of endometriotic lesions and the alleviation of associated symptoms. The impairment of AKT, ERK1/2, and mTOR pathways reduces cell proliferation and survival, further highlighting dienogest’s multifaceted role in managing endometriosis. Tucci et al. and Samare Najaf et al. demonstrated that the overactivation of PI3K-Akt-mTOR signaling pathway has been confirmed in endometriosis [31,32]. Supressing mTOR signaling pathway suppress ectopic endometrial cell migration. Therefore, dienogest might block mTOR signaling pathway and mitigate endometriosis.

In addition to reducing lesion size, dienogest effectively alleviates pain associated with endometriosis. It is essential to note that the severity of pain symptoms in endometriosis does not always correlate with the extent of lesions. Though It is reported that pain symptom scores in deep dyspareunia and lower back pain of patients is associated with different endometriosis lesions [33]. One of the key mechanisms through which dienogest achieves pain relief is by inhibiting the expression of nerve growth factor (NGF) in human endometrial epithelial cells (hEECs) [34]. NGF is a critical mediator of pain in endometriosis, often upregulated by inflammatory cytokines such as tumor necrosis factor-α (TNF-α) and interleukin-1β (IL-1β) [35]. Dienogest inhibits NGF mRNA and protein production in hEECs expressing PR-A and PR-B, thereby reducing the neural sensitization and pain signaling pathways involved in endometriosis [36]^.^ The significant reduction in the diameter and volume of endometriomas after 12 months of treatment underscores dienogest’s efficacy as a therapeutic option for managing large ovarian endometriomas.

Dienogest has emerged as an effective treatment for pain associated with endometriosis. While side effects such as irregular vaginal bleeding, weight gain, headaches, acne, hair loss, mood swings, and breast tenderness have been noted (20), weight gain was identified as the most prevalent adverse reaction over a year of treatment. It is hypothesized that progestogens increase appetite, contributing to weight gain during treatment. Patients were informed of possible side effects beforehand, and the majority reported tolerable experiences, indicating the drug’s suitability for long-term use.

## 5. Conclusion

In summary, this single-center, prospective cohort study has provided real-world clinical data from China on the treatment of large ovarian endometriosis cysts with dienogest. The study demonstrated that dienogest treatment of ovarian endometriosis cysts with a maximum diameter of ≥4 cm could alleviate pain, reduce cyst size, show a more significant reduction, treat endometrial polyps, improve anemia, and protect ovarian function. Our research provides new insights into using Dienogest for patients with larger endometriomas who prefer not to undergo surgery, offering a novel therapeutic approach.This study uniquely examines the effects of Dienogest on uterine size, endometrial thickness, and uterine fibroids during treatment. However, our research is a prospective, single-arm study conducted in a real-world clinical setting, which lacks a control group. The study involves a relatively small number of participants and a short follow-up period. Additionally, thought these findings suggested that dienogest showed a protective effect on ovarian reserve function, Opting for pharmacological treatment could significantly contribute to preserving female fertility. Nevertheless, the potential of dienogest to improve pregnancy rates and live birth rates post-discontinuation, when women desire to conceive, remains to be determined by clinical studies to warrant further attention.

## Data Availability

The data that support the findings of this study are available upon reasonable request from the corresponding author, CP.
